# Supplementary material for: WRKY22 Transcription Factor from Iris laevigata Regulates Flowering Time and Resistance to Salt and Drought
Source: Plants (Basel). 2024 Apr 25;13(9):1191. doi: 10.3390/plants13091191 (PMC11085594; doi:10.3390/plants13091191)
Supplement: Supplementary file 1 [file plants-13-01191-s001.zip › Supplemental Figures.pdf]

# Supplemental Figures

# Supplemental Figure S1.

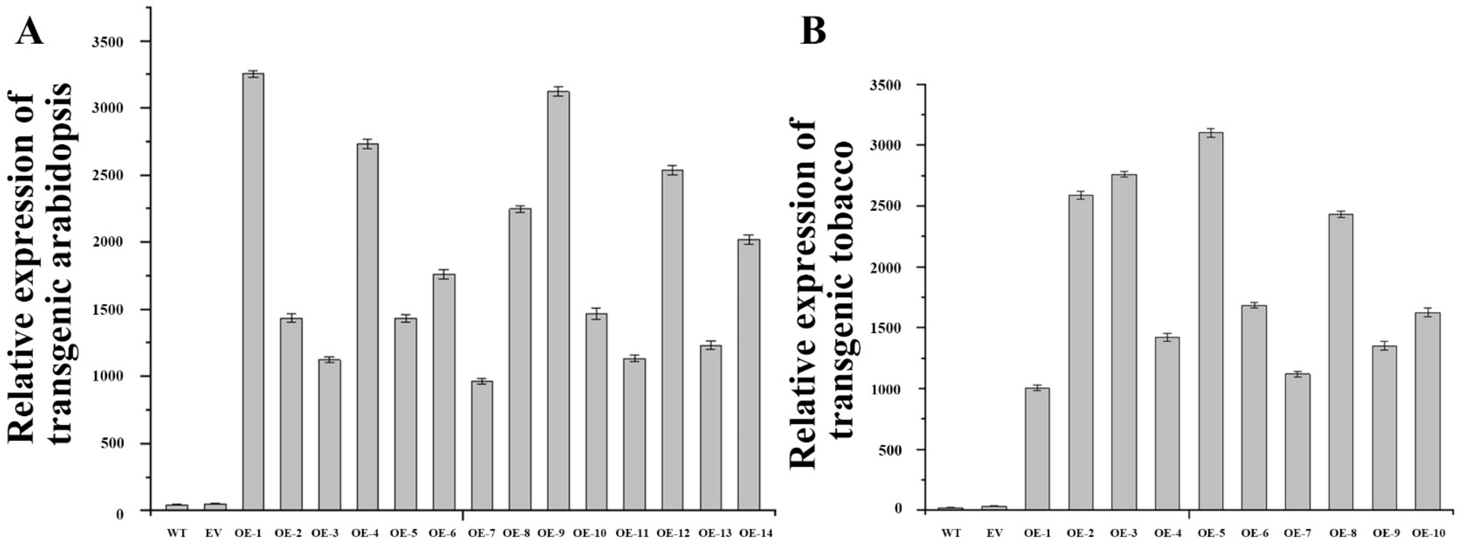


**Supplemental Figure 1.** Gene expression profile of *IlWRKY22* transgenic T3 generation plants. **A** Relative expression of *IlWRKY22* in fourteen T3 transgenic *A. thaliana* lines. **B** Relative expression of *IlWRKY22* in ten T3 transgenic *N. tabacum* lines.

# Supplemental Figure S2.

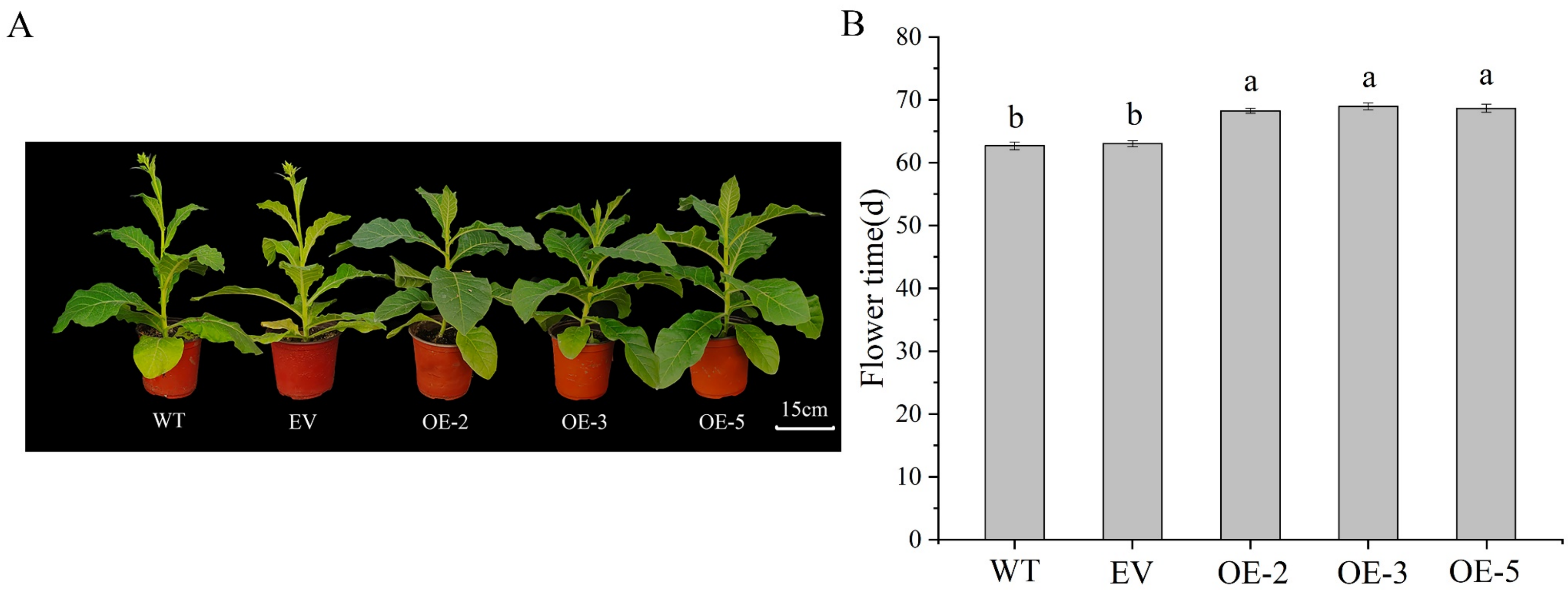


**Supplemental Figure 2.** Phenotypes of transgenic *N. tabacum* overexpressing *IlWRKY22*. **A** Phenotypic observation of transgenic *N. tabacum* overexpressing *IlWRKY22*. Scale bar was 15 cm. **B** Flowering time (opening of the first flower). Bar graph data are Mean ± SD, marked with different lowercase letters to indicate significant differences ($p < 0.05$). All these experiments were performed in triplicate.

# Supplemental Figure S3.

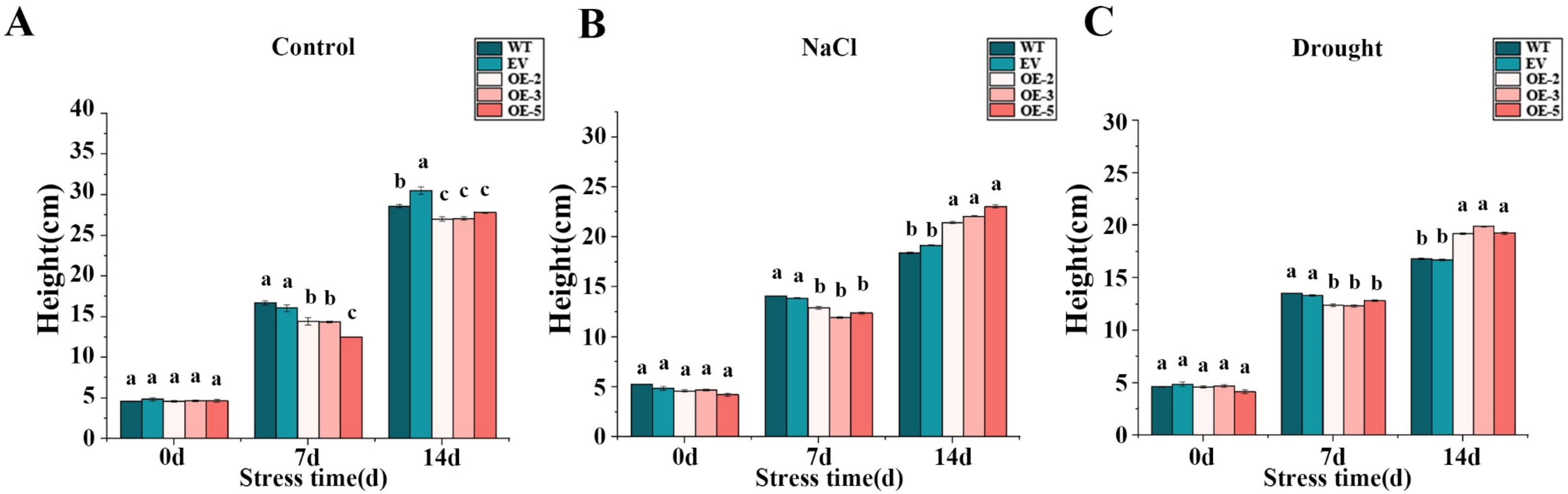


**Supplemental Figure 3.** Overall plant height under salt and drought stress at 0, 7 and 14 dpt. **A** Plant height observation of WT, EV, and OE lines of *N. tabacum* at 0, 7, and 14 days in the normal growth state. **B** Plant height observation of WT, EV, and OE lines of *N. tabacum* at 0, 7, and 14 days under NaCl stress at 300mM. **C** Plant height observation of WT, EV, and OE lines of *N. tabacum* at 0, 7, and 14 days under natural drought stress. Bar graph data are Mean ± SD, marked with different lowercase letters to indicate

significant differences ($p < 0.05$). All these experiments were performed in triplicate.
