# Supplementary material for: WRKY22 Transcription Factor from Iris laevigata Regulates Flowering Time and Resistance to Salt and Drought
Source: Plants (Basel). 2024 Apr 25;13(9):1191. doi: 10.3390/plants13091191 (PMC11085594; doi:10.3390/plants13091191)
Supplement: Supplementary file 1 [file plants-13-01191-s001.zip › Supplemental Tables.pdf]

Supplementary Material

Table S1. Primer sequence and application

**Name**	**Forward / Reverse primer sequence（5'-3'）**	**Temperature/℃**	**Application**
IlWRKY22	F:CTCCCAACAAACTCTCTCCCTAG	56℃	PCR amplification reaction
IlWRKY22	R:CAACAAAATAATCAGCTTGCCCC		
AtActin	F:CTCCTTTGTTGCTGTTGACTAC	60℃	Fluorescence quantitative reference genes
AtActin	R:GCACAATGTTACCGTACAGATC		
IlWRKY22	F:GATTGGAGTTGAGATGGGCTT	56℃	Fluorescence quantitative PCR reaction
IlWRKY22	R:ACTGTATAGTGGTGCTGAGGC		
IlWRKY22-BamHI	F:TTGATACATATGCCCGTCGACTTCTCTCCATCCCCTCTTT	60℃	PCR amplification reaction
IlWRKY22-SalI	R:CCCTTGCTCACCATGGATCCGCTCCCACCACCGGCAACGG		
CO	F:CACAGGTGAATACAGTCAACACC	60℃	Fluorescence quantification
CO	R:CCATGGATGAAATGTATGCGTTATGG		
GA20OX	F:CGGTTTTGCGACGACATGAG	60℃	Fluorescence quantification

GA20OX	R:TAGCCCCAGAAGCTCCATGA		
VRN1	F:CTGAGGGTCCCAGATAAGTTTG	60℃	Fluorescence quantification
VRN1	R:GTCAGCTTTCCTTAGTCCTACAC		
SPL3	F:CTCATGTTCGGATCTCTGGTC	60℃	Fluorescence quantification
SPL3	R:TTTCCGCCTTCTCTCGTTGTG		
FCA	F:GCTCTTGTCGCAGCAAACTC	60℃	Fluorescence quantification
FCA	R;GATCCAGCCCACTGTTGTTTAC		
SVP	F:GAAGAGAACGAGCGACTTGG	60℃	Fluorescence quantification
SVP	R:GAGCTCTCGGAGTCAACAGG		
SOC1	F:GATCGAGTCAGCACCAAACC	60℃	Fluorescence quantification
SOC1	R:TCCTATGCCTTCTCCCAAGA		
TPS1	F:ATTGGCATAGATTCTGATCGGT	60℃	Fluorescence quantification
TPS1	R:TCAAGACGATCAACACCTAACA		
FLC	F:AGCCAAGAAGACCGAACTCA	60℃	Fluorescence quantification

FLC	R:AGCTTCTGCTCCCACATGAT		
NtHAK1	F:ATCCACACCGAGCTTGTTTCAGGA	60℃	Fluorescence quantitative PCR reaction
NtHAK1	R:TGGGTCCAATTCTTCCCACCAAGA		
NtSOS1	F:GCGTGCTTATTTCCACCTTTTG	60℃	Fluorescence quantitative PCR reaction
NtSOS1	R:TTTGATGACGGCTCCCCAGT		
NtPMA4	F:TTTCCCGAGCACAAGTATGA	60℃	Fluorescence quantitative PCR reaction
NtPMA4	R:GGTAACCTCCAAGAACAACAC		
NtSOD	F:CTCCTACCGTCGCCAAAT	60℃	Fluorescence quantitative PCR reaction
NtSOD	R:GCCCAACCAAGAGAACCC		
NtCAT	F:AGGTACCGCTCATTCACACC	60℃	Fluorescence quantitative PCR reaction
NtCAT	R:AAGCAAGCTTTTGACCCAGA		
NtPOD	F:CCTCAGCTTCAAGCATTATGTCCA	60℃	Fluorescence quantitative PCR reaction
NtPOD	R:ACCTTTGTAGAAGCATCGGTCCAC		
NtActin	F:CGGAATCCACGAGACTAACATACAAC	60℃	

NtActin	R:GGTGCTGAGGGAAGCCA AGATA	Fluorescence quantitative PCR reaction

Table S2. Functional prediction of *WRKY* transcription factor family members in *I. laevigata*

**Group**	***A. thaliana***	***I. laevigata***	**Function**
I	*AtWRKY1*<br>*AtWRKY2*<br>*AtWRKY3*<br>*AtWRKY4*<br>*AtWRKY25*<br>*AtWRKY26*<br>*AtWRKY32*<br>*AtWRKY33*<br>*AtWRKY34*<br>*AtWRKY44*<br>*AtWRKY58*	isoform 3308<br>isoform 275946<br>isoform 813581<br>isoform 336911<br>isoform 460429<br>isoform 71297<br>isoform 722579<br>isoform 629169<br>isoform 239732<br>isoform 238068<br>isoform 687755<br>isoform 416995<br>isoform 342824<br>isoform 439137<br>isoform 312565<br>isoform 658241<br>isoform 7202<br>isoform 6445<br>isoform 232908<br>isoform 564486	They are involved in disease resistance, abiotic stress (salt, cold, heat), senescence and some developmental processes (pollen development, seed coat development). A response to a light stimulus.

Group	*A. thaliana*	*I. laevigata*	Function
		isoform 298714<br>isoform 319525	
IIa	*AtWRKY40*<br>*AtWRKY60*	isoform 49561<br>isoform 305653<br>isoform 347489<br>isoform 63513<br>isoform 684413	A defensive response to bacteria or fungi.
IIb	*AtWRKY6*<br>*AtWRKY9*<br>*AtWRKY31*<br>*AtWRKY36*<br>*AtWRKY42*<br>*AtWRKY47*<br>*AtWRKY72*	isoform 358692<br>isoform 56218<br>isoform 320236<br>isoform 40279<br>isoform 478449<br>isoform 303256<br>isoform 14002	Defense responses to bacteria, fungi, oxidative stress; expression of genes involved in cold, leaf senescence. Root development.
IIc	*AtWRKY8*<br>*AtWRKY12*<br>*AtWRKY13*<br>*AtWRKY23*<br>*AtWRKY24*<br>*AtWRKY28*	isoform 735698	They are involved in disease resistance (bacteria, fungi), abiotic stress (salt, cadmium, drought) , senescence and some developmental processes (lignin synthesis, pollen development, florescence regulation) Auxin transport.

Group	*A. thaliana*	*I. laevigata*	Function
	AtWRKY43<br>*AtWRKY48*<br>*AtWRKY49*<br>*AtWRKY50*<br>*AtWRKY51*<br>*AtWRKY56*<br>*AtWRKY57*<br>*AtWRKY59*<br>*AtWRKY71*<br>*AtWRKY75*		
IId	*AtWRKY7*<br>*AtWRKY11*<br>*AtWRKY15*<br>*AtWRKY21*<br>*AtWRKY39*<br>*AtWRKY74*	isoform 352866<br>isoform 287969<br>isoform 241617<br>isoform 279981<br>isoform 327411<br>isoform 297487<br>isoform 410198<br>isoform 41879<br>isoform 567039<br>isoform 513126<br>isoform 286734<br>isoform 305537<br>isoform 596689	They are involved in plant disease resistance (bacteria, fungi), senescence (apoptosis) and some developmental processes (flower development, florescence regulation), regulation of jasmonic acid signaling pathway, response to light stimulation, calmodulin binding, reaction of salicylic acid.

Group	*A. thaliana*	*I. laevigata*	Function
IIe		isoform 561421	They are involved in disease resistance (bacteria), senescence and some developmental processes (epidermis development, flower development, plant organ and root morphogenesis, florescence regulation), auxin transport.
		isoform 494831	
	AtWRKY14	isoform 588901	
	AtWRKY16	isoform 420051	
	AtWRKY22	isoform 234765	
	AtWRKY27	isoform 635473	
	AtWRKY29	isoform 51755	
	AtWRKY35	isoform 422731	
		isoform 304457	
III	*AtWRKY30*	isoform 507977	Involved in plant disease resistance (bacteria, fungi), abiotic stress (ozone, temperature, drought), senescence and some developmental processes (lateral root development, leaf development); response to salicylic acid, regulation of abscisic acid signaling pathway. Regulates brassinosteroid, ethylene and jasmonic acid-mediated signaling pathways.
	AtWRKY38	isoform 31149	
	AtWRKY41	isoform 614906	
	AtWRKY46	isoform 69705	
	AtWRKY53	isoform 38178	
	AtWRKY54	isoform 487979	
	AtWRKY55	isoform 645693	
	AtWRKY63	isoform 629546	
	AtWRKY64	isoform 44179	
	AtWRKY66	isoform 481801	
	AtWRKY70	isoform 20352	

Table S3. Abbreviations

**Name**	**Online addresses**
CO	constans
FLC	flowering locus C
CDF	cycling dof factors
FT	flowering locus T
SOC1	suppressor of overexpression of constans1
ABA	abscisic acid
GA20OX	GA-20 oxidase
SPL3	squamosa promoter binding protein like3
WT	wild type
NJ	neighbor-joining
VRN1	vernalization1
FCA	flowering control local A
TPS1	trehalose-6-phosphate synthase1
SVP	short vegetative phase
Pn	net photosynthetic rate
Gs	stomatal conductance
Ci	intercellular CO_2 concentration
Tr	transpiration rate

Chl content	chlorophyll content
dpt	days post-treatment
ROS	reactive oxygen species
MDA	malondialdehyde
SOD	superoxide dismutase
CAT	catalase
POD	peroxidase
DAB	3,3 '-diaminobenzidine tetrahydrochloride
NCBI	National Center for Biotechnology Information
NBT	p-Nitro-Blue tetrazolium chloride
